# Tbx18 and the generation of a biological pacemaker. Are we there yet?

**DOI:** 10.1016/j.yjmcc.2016.06.006

**Published:** 2016-08

**Authors:** Thomas Brand

**Affiliations:** Developmental Dynamics, Heart Science Center, National Heart and Lung Institute, Imperial College London, United Kingdom

**Keywords:** Sinus node, Gene program, Calcium clock, Membrane clock, Transcriptional control, repressor, Biological pacemaker

A group of approximately 10,000 cells in the sinoatrial node (SAN), which is located at the entry of the right superior caval vein into the right atrium, is responsible for regular heart beating under different physiological conditions [1]. While the SAN is reliably working for most of our life, in the elderly, sick sinus syndrome (SSS), or sinus node dysfunction (SND) is prevalent [2] and responsible for 30 to 50% of all electronic pacemaker implantations [3]. Moreover, SSS is also often associated with the development of atrial fibrillation [4]. A fraction of the SSS cases is familial and has a genetic basis. Moreover, several null mutants in mice display bradyarrhythmias during postnatal life and may serve as animal models of SSS. SSS is also the result of intrinsic and extrinsic factors causing fibrosis and other structural impairments [4]. Treatment options are currently limited to the implantation of an electronic pacemaker. While being effective, the electronic pacemaker is insensitive to hormonal stimulation, has the hazards of infections, and possibly the replacement of the pacemaker may be required. Thus, there is demand to develop a biological pacemaker, which may overcome these problems.

The SAN makes up an extensive area of the intercaval myocardium. Transitory cells are surrounding the primary nodal cells, which display both pacemaking abilities and fast conduction [5]. The SAN is densely innervated by the autonomic nervous system. It is separated from the working myocardium by connective tissue, which functions as electrical insulation and may protect the SAN from getting hyperpolarized. Optical mapping revealed the presence of exit pathways in the human SAN via which impulses are propagated into surrounding atrial muscle [6]. SAN myocytes are poorly coupled due to the presence of small amounts of slow conducting gap junctions consisting of connexin (Cx) 45 and Cx30.2 [7]. There are significant morphological differences between SAN myocytes and chamber myocytes (Fig. 1A). Sinus node cells have a small cell body and long thin cellular extensions [8]. The myofibrillar content in SAN myocytes is low, they lack T-tubules, but contain larger amounts of caveolae in comparison to atrial myocytes [9], [10].

The ability of the SAN cells to act as the pacemaker of the heart depends on two oscillatory mechanisms, the Ca^2 +^-clock and the membrane clock that are interlinked by the release of calcium from the sarcoplasmatic reticulum and drive a slow depolarizing sodium current via the exchange of calcium by the sodium calcium exchanger (NCX) [11]. The Ca^2 +^-clock components include the ryanodine receptor (Ryr2), the SR Ca^2 +^-ATPase (SERCA II) and the Ca^2 +^-storage proteins of the sarcoplasmatic reticulum. The membrane clock components of SAN cells include, apart from NCX, voltage-gated calcium channels (T- and L-type) and the hyperpolarization-activated cyclic nucleotide-gated channels (HCN1, HCN2 and HCN4) [12]. Importantly, the sodium channel SCN5A is not expressed in the SAN, while brain-type sodium channels are present [13]. Therefore, the upstroke velocity of the action potential of SAN cells is significantly smaller and the action potential has a lower amplitude than in chamber myocytes (Fig. 1B). The ability of SAN cells to start depolarization at hyperpolarization is due to the funny current (I_f_), which is specific to nodal cells and driven by HCN channels [14]. The pacemaking ability of the SAN is therefore due to a complex anatomy, a specific histology and myocyte morphology and is based on a unique set of ion channels interlinked in a complex manner.

In the early embryo, the linear heart tube consists of primitive myocytes which all display automacity. The newly recruited cardiac myocytes at the venous pole are acting as dominant pacemaker [15]. During heart looping, regions at the outer curvature of the heart tube start to proliferate and acquire a chamber-specific gene expression program, which includes high conductance type connexins (Cx40 and Cx43) and the sodium channel SCN5A [16]. The chamber-specific gene expression program is actively repressed in the myocytes of the sinus venosus (SV) through a complex set of transcriptional repressors (Fig. 1C). In avian embryos, shortly after gastrulation, SAN cells have their origin in the right lateral plate mesoderm just posterior of the heart field [17]. It is unknown when SAN cells become specified in mammalian embryos. The sinus venosus myocardium, which makes up a large part of the SAN starts to differentiate from embryonic day (E) 9.5 in the mouse [18] and the SAN can morphologically be identified from embryonic day (E) 11.5 [19].

In mice, the entire early heart tube expresses the transcription factor *Nkx2.5*. However, the newly added SV myocardium at the venous pole is derived from a lineage, which expresses *Tbx18* and lacks *Nkx2.5*. Mice that lack *Tbx18* fail to develop the SAN head and display a malformed SV. Despite this, the pacemaking is not affected in the null mutant, suggesting that *Tbx18* is responsible for morphological aspects of SAN formation but dispensable for its functional development [20].

*Tbx3* is also expressed in the SAN [21] and represses the transcriptional program of chamber myocardium. In mice lacking *Tbx3,* the SAN acquires a gene expression program normally found in the atrium [21]. Forced expression of *Tbx3* in fetal atria causes repression of chamber-specific genes, upregulation of SAN-specific genes and display ectopic automacity [21]. Expression of *Tbx3* in adult atria suppressed the working myocardium gene program, without ectopic expression of SAN genes [22]. Forced expression in embryonic stem (ES) cells effectively induced myocytes with functional pacemaker-like abilities and morphological features of SAN cells [23]. These data suggest that the ability to transform working myocardium into pacemaker tissue by *Tbx3* is gradually lost during cardiac maturation.

The short stature homeobox transcription factor 2 (Shox2), another repressor of the chamber gene expression program, acts via repression of *Nx2.5* while activating expression of the transcription factor islet 1 (*Isl1*). Loss of *Shox2* causes hypoblastic SAN development [24]. Forced expression in cultured ES cells induces a pacemaker-like phenotype [25]. The transcription factor Isl1 acts downstream of *Shox2* [26]. While *Isl1* is only transiently expressed in cardiac mesodermal progenitors and lost after differentiation, it is maintained in SAN myocytes in both the embryonic and adult heart [27]. Isl1 is required for the proliferation of SAN precursors. SAN-specific ablation of *Isl1* causes embryonic lethality [28] and is accompanied by a loss of expression of *Tbx3*, *Shox2,* as well as ion channels important for cardiac pacemaking. Forced expression of *Isl1* in ES cells is able to upregulate SAN-specific genes and to suppress chamber-specific genes [29].

The SAN develops only on the right side of the embryo. This unilaterality of SAN development is achieved through direct repression of *Shox2* by *Pitx2* and by the upregulation of two microRNAs, which are able to suppress SAN genes such as *Tbx3* and *Shox2* [30]. In *Pitx2*-deficient embryos, SAN development occurs bilaterally [31].

In order to identify the most potent transcription factor with the ability to convert working myocytes into SAN cells, a panel of transcription factors (Shox2, Tbx3, Tbx5 and Tbx18, Tbx20) has been virally expressed in neonatal ventricular myocytes [32]. Only Tbx18 caused an increase in the spontaneous beating rate. Moreover, *Hcn4* was induced, the action potential displayed SAN-like properties and the cells displayed SAN-like cell morphology. Tbx18 was also able to suppress *Cx43* expression [33]. Gene transfer of *Tbx18* was also attempted with adult pig hearts having a complete heart block [34]. Surprisingly, cardiac automacity and independence of an implanted electronic pacemaker was observed. Cells in the vicinity of the injection site displayed upregulation of *Hcn4* and a downregulation of chamber-specific marker genes. Moreover, the *Tbx18*-expressing cells displayed altered cell morphology. This study represents the first successful induction of a biological pacemaker by viral gene delivery.

In a recent elegant study from the Kispert group [35], the ability of *Tbx18* to convert working myocardium into pacemaker cells was further studied. The authors utilized a conditional Cre/loxP-based transgenic approach to express *Tbx18* in atrial and ventricular chamber myocardium. In Myh6-Cre/Tbx18 hearts expression of the transgene was detectable at E12.5 and expression became more robust at the fetal stage. Ectopic expression of *Tbx18* caused right ventricular hypoplasia, atrial dilatation and ventricular septal defects. However, no ectopic expression of SAN-specific genes was observed in the transgenic atria or ventricles. Several chamber-specific genes (including *Gj5*, *Scn5A, Kcnj2* and *Kcnj3*) were suppressed after *Tbx18* expression. A transcriptome analysis revealed an ectopic expression of *Nppa* in the ventricles and of ventricular marker genes (*Mlc2v*, *Myh7*, and *Myl2*) in the atria. Significantly, *Pitx2*, which is normally expressed in the left atrium was suppressed. Another Cre-driver, Tagln-Cre was also utilized to further validate these results. However, also in this case no ectopic expression of SAN-specific genes was observed, but again aberrant cardiac morphology and altered expression of chamber-specific genes were present.

These data are in line with the results of the *Tbx18* null mutant, which displayed structural abnormalities, without affecting pacemaking [20]. However, these results do not fully agree with the recent finding that *Tbx18* is able to induce a SAN gene program when virally overexpressed [32]. Both studies found that *Tbx18* is able to suppress chamber-specific gene expression.

An explanation for the different outcomes of the two studies might be the use of two different animal models. These two species might differentially utilize *Tbx3* and *Tbx18*. It is theoretically possible that SAN-specific gene expression is under the control of *Tbx18* in the porcine heart, while it is controlled by *Tbx3* in the mouse. Another difference lies in the experimental design: a transgenic approach was used by Greulich et al. [35] and *Tbx18* was expressed in the fetal heart, while viral infection of the adult porcine heart was utilized in the other study [34]. Thus, the levels of transgene expression are probably different and most importantly the developmental time at which *Tbx18* was expressed differed in the two studies.

Nonetheless, the presence of atrial and ventricular pathologies and the loss of expression of vital genes, such as *Pitx2*, after ectopic expression of *Tbx18* in the mouse heart should be taken seriously. Therefore, further research is required to rule out any serious side effects, which might accompany the induction of a SAN gene program in the chamber myocardium.

In conclusion, more work is required, in particular in the porcine and human heart, to establish whether there are species-specific differences in the transcriptional control of SAN specification. While the results of the viral overexpression of *Tbx18* are exciting and highly encouraging towards the goal of developing a biological pacemaker, there is now evidence that there might be the risk of cardiac pathologies as the result of ectopic *Tbx18* expression.

## Disclosures

None.

## Figures and Tables

**Fig. 1:**
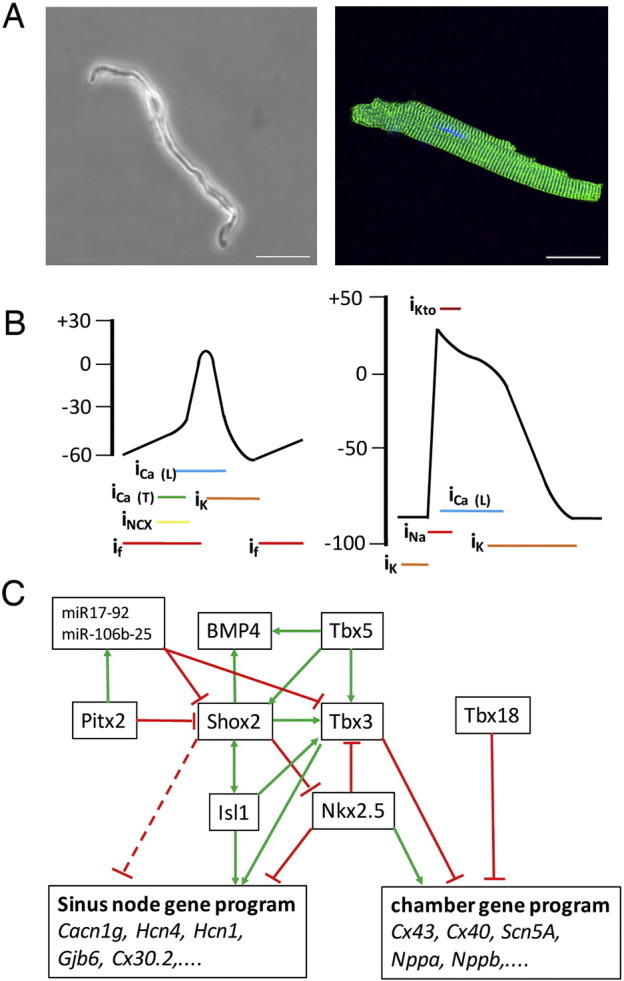
Comparison of sinotrial pacemaker cells and chamber myocytes. (A) Morphological comparison of a SAN myocyte (phase contrast) and a ventricular myocyte, which was immunochemically stained for Ryr2 expression. Bar represents 25 μm. (B) Comparison of the action potentials of a SAN myocyte (left) and a ventricular myocyte (right). (C) Transcriptional network, which determines SAN development. Panel C was adapted from [15].
